# Cortisol excess in chronic kidney disease – A review of changes and impact on mortality

**DOI:** 10.3389/fendo.2022.1075809

**Published:** 2023-01-17

**Authors:** Michael S. Sagmeister, Lorraine Harper, Rowan S. Hardy

**Affiliations:** ^1^ Institute for Metabolism and Systems Research, University of Birmingham, Birmingham, United Kingdom; ^2^ Renal Medicine, University Hospitals Birmingham NHS Foundation Trust, Birmingham, United Kingdom; ^3^ Institute for Applied Health Research, University of Birmingham, Birmingham, United Kingdom; ^4^ Research into Inflammatory Arthritis Centre Versus Arthritis, Institute of Inflammation and Ageing, University of Birmingham, Birmingham, United Kingdom; ^5^ Institute of Clinical Science, University of Birmingham, Birmingham, United Kingdom

**Keywords:** subclinical hypercortisolism, steroid metabolism, 11beta-HSD, renal replacement therapy, human, chronic disease, adrenal function, circadian rhythm

## Abstract

Chronic kidney disease (CKD) describes the long-term condition of impaired kidney function from any cause. CKD is common and associated with a wide array of complications including higher mortality, cardiovascular disease, hypertension, insulin resistance, dyslipidemia, sarcopenia, osteoporosis, aberrant immune function, cognitive impairment, mood disturbances and poor sleep quality. Glucocorticoids are endogenous pleiotropic steroid hormones and their excess produces a pattern of morbidity that possesses considerable overlap with CKD. Circulating levels of cortisol, the major active glucocorticoid in humans, are determined by a complex interplay between several processes. The hypothalamic-pituitary-adrenal axis (HPA) regulates cortisol synthesis and release, 11β-hydroxysteroid dehydrogenase enzymes mediate metabolic interconversion between active and inactive forms, and clearance from the circulation depends on irreversible metabolic inactivation in the liver followed by urinary excretion. Chronic stress, inflammatory states and other aspects of CKD can disturb these processes, enhancing cortisol secretion *via* the HPA axis and inducing tissue-resident amplification of glucocorticoid signals. Progressive renal impairment can further impact on cortisol metabolism and urinary clearance of cortisol metabolites. Consequently, significant interest exists to precisely understand the dysregulation of cortisol in CKD and its significance for adverse clinical outcomes. In this review, we summarize the latest literature on alterations in endogenous glucocorticoid regulation in adults with CKD and evaluate the available evidence on cortisol as a mechanistic driver of excess mortality and morbidity. The emerging picture is one of subclinical hypercortisolism with blunted diurnal decline of cortisol levels, impaired negative feedback regulation and reduced cortisol clearance. An association between cortisol and adjusted all-cause mortality has been reported in observational studies for patients with end-stage renal failure, but further research is required to assess links between cortisol and clinical outcomes in CKD. We propose recommendations for future research, including therapeutic strategies that aim to reduce complications of CKD by correcting or reversing dysregulation of cortisol.

## 1 Introduction

**Figure 1:**
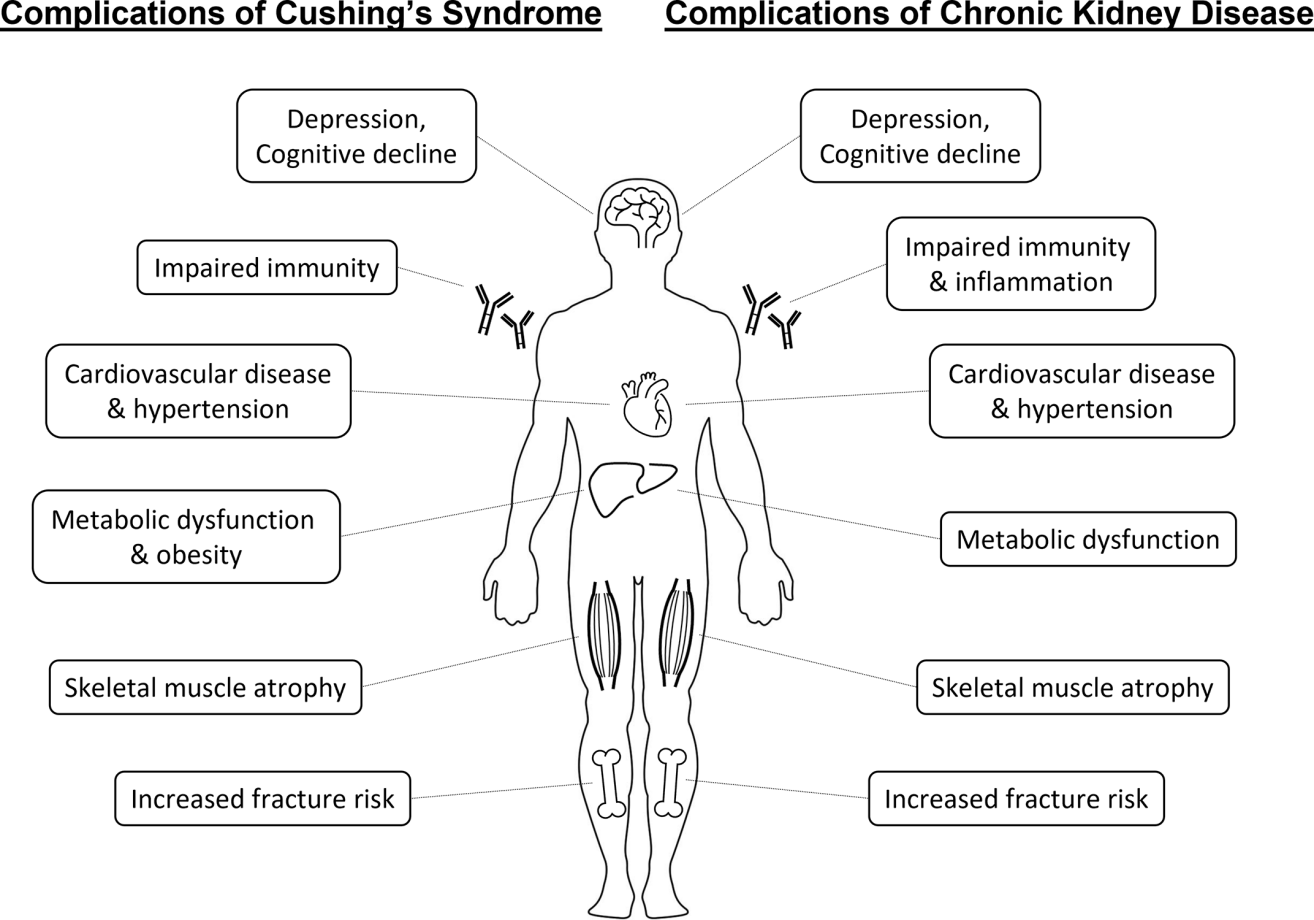
Similarities between systemic complications of Cushing’s syndrome and chronic kidney disease.

Chronic kidney disease (CKD) affects 2.6 million people in the United Kingdom (6.1% of the population), with over 29,000 requiring regular dialysis treatment for end-stage kidney failure (0.04% of the population) (1, 2). Patients with CKD have increased risk of cardiovascular disease and death (3). They also commonly develop a characteristic range of complications including hypertension, cardiovascular events, insulin resistance, dyslipidemia, sarcopenia, osteoporosis and fractures, infections and mood or sleep disturbances (4) (**Figure 1**). However, fundamental gaps in our knowledge remain about the mechanisms underpinning increased mortality and morbidity in chronic renal impairment, limiting the efficacy of therapeutic intervention.

Glucocorticoids represent a family of endogenous pleotropic steroid hormones that are essential to life and regulate a wide array of critical physiological processes. The major active endogenous glucocorticoid in humans, cortisol, plays a crucial role mediating the body’s response to a diverse array of stressors (5, 6). Cortisol acts through binding to the glucocorticoid receptor, which is ubiquitously expressed and mediates a wide range of cell-specific effects (7). These in turn influence blood pressure and circulation, glucose homeostasis, immune function, metabolism, tissue remodelling and cognition (5). The homeostasis of cortisol is itself dynamic, involving multiple mechanisms. These include central regulation of adrenal gland synthesis and secretion *via* the hypothalamic- pituitary-adrenal axis (HPA), systemic and peripheral tissue metabolism (both activating and inactivating), terminal metabolic inactivation in the liver and excretion of metabolites in urine (8–10).

Both glucocorticoid deficiency (adrenal insufficiency) and glucocorticoid excess (Cushing’s syndrome) are themselves causes of significant morbidity and mortality (11, 12). Even a mild degree of persistent elevated cortisol exposure has a profound impact on mortality. Overall mortality and in particular cardiovascular mortality remains elevated in patients with treated Cushing’s disease 10 years after remission compared to the general population (13). Slight increments in hydrocortisone replacement dose for adrenal insufficiency translate into significantly increased mortality related to cardiovascular, respiratory and malignant disease (14). And in patients with adrenal incidentalomas, evidence of elevated adrenal cortisol output in the absence of clinical features of Cushing’s syndrome predicts mortality related to cardiovascular disease and infection (15). Of interest, glucocorticoid excess possesses considerable overlap with the pattern of complications observed in CKD, e.g. hypertension, elevated cardiovascular risk, insulin resistance, dyslipidaemia, sarcopenia, osteoporosis, immune dysfunction or mood disturbances (**Figure 1**). This warrants consideration whether these similarities are co-incidental or share underlying biological links.

In this review, we have summarised the literature characterising the alterations in endogenous glucocorticoid regulation in patients with chronic kidney disease (see Box 1 for search methodology). We subsequently explore the evidence for disturbances in cortisol regulation as a mechanistic driver of excess mortality and morbidity in CKD. Finally, this review will consider the implications for future therapeutic strategies that aim to correct or reverse alterations in glucocorticoid signalling in patients with CKD.

**Box 1: Literature Search Methodology**

The OVID-Medline database was searched on 2^nd^ May 2022 for any randomised or non-randomised trial, observational study or experimental study that provided information on endogenous cortisol regulation in humans with any level of reduced kidney function (see supplementary materials for full list of search terms). Studies involving children, acute illness, organ transplantation or exogenous steroid administration were excluded to reduce undue heterogeneity and biases. Case reports and publications unavailable in English were also excluded. Search results were filtered for studies published since 1995. The rationale for this included significant advances in management of CKD that lower physiological stress (e.g. erythropoietin stimulating agents, dialysis membranes with greater biocompatibility) and inferior specificity of historic assays for cortisol. A few exceptions were made to discuss studies published before 1995, when no alternative sources of information were available and the findings were critical to understanding changes in cortisol regulation in CKD.

## 2 Physiological regulation of circulating glucocorticoids

**Figure 2:**
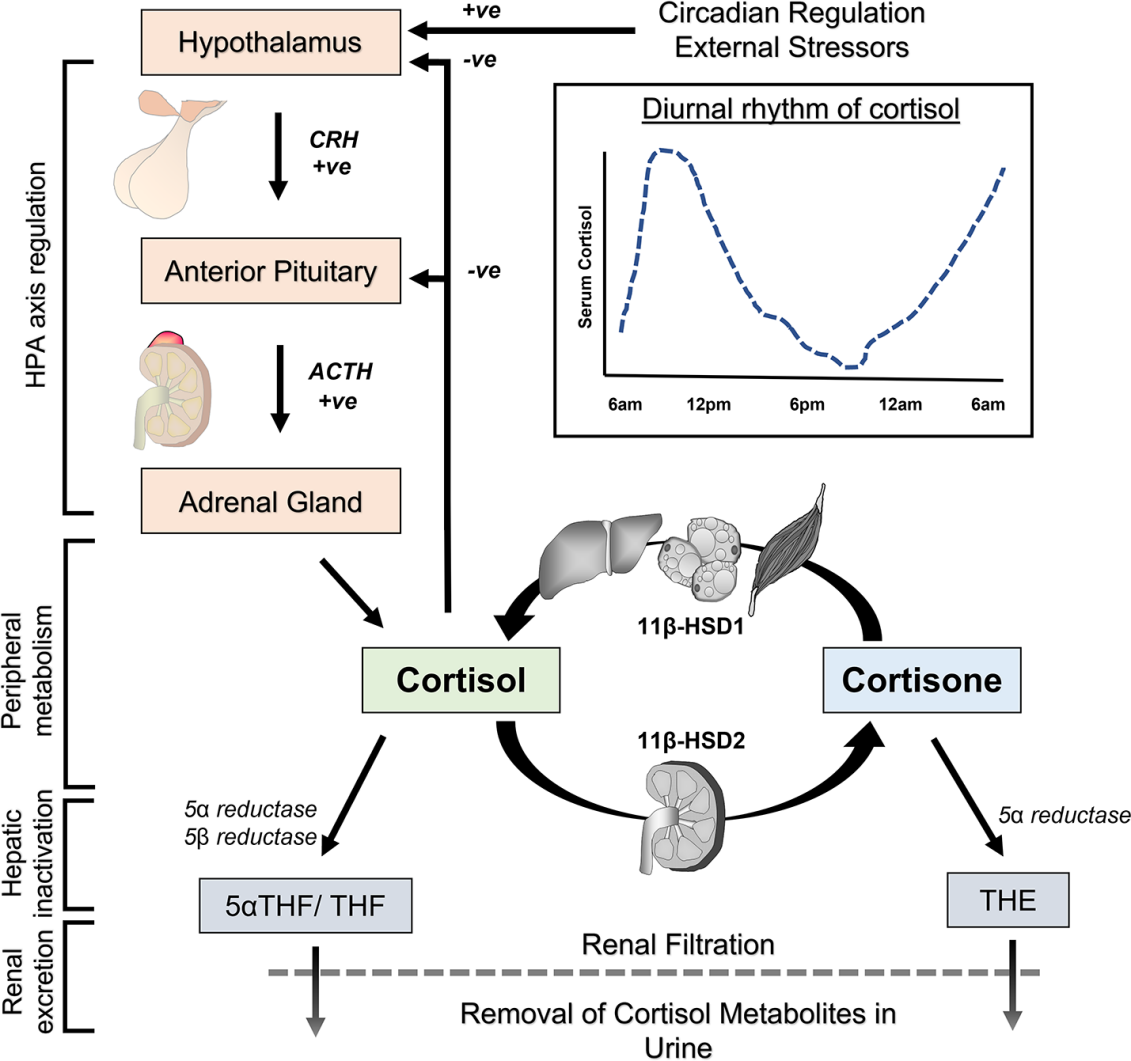
Pathways for physiological regulation of cortisol in the body. The hypothalamic-pituitary-adrenal (HPA) axis controls cortisol synthesis in the adrenal gland and its release into the circulation. The activity of the HPA axis is set by the circadian clock, negative feedback from circulating cortisol, external stressors and other factors. Cortisol and inactive cortisone are interconverted by 11β-hydroxysteroid dehydrogenase (11 β-HSD) enzymes for reversible activation and inactivation in peripheral tissues. Liver, fat and skeletal muscle are predominant sites of cortisol activation by 11 β-HSD1, while kidney is the predominant site for reversible cortisol inactivation by 11 β-HSD2. Terminal inactivation of cortisol occurs in the liver through enzymatic reduction to tetrahydrocortisol (THF), 5α-tetrahydrocortisol (5α-THF) and tetrahydrocortisone (THE). These metabolites are ultimately removed from the body through filtration in the kidneys and excretion in urine.

Levels of cortisol in circulation vary significantly throughout the day. Cortisol levels exhibit a circadian rhythm under regulation of the hypothalamic circadian clock (6, 8). Levels are typically highest in the morning, declining throughout the day and reaching a nadir around midnight (**Figure 2**). On top of this circadian rhythm, cortisol levels exhibit ultradian fluctuations. This means cortisol levels rise and fall on time scales significantly shorter than 24 hours. Reasons for ultradian fluctuations include pulsatile nature of cortisol release from the adrenal glands, increments in cortisol following meals or with stress and other factors. Consequently, levels of cortisol in blood for the same individual can vary significantly over 30 minutes.

Systemic levels of cortisol are under the control of a homeostatic mechanism involving the hypothalamus, anterior pituitary gland and adrenal glands (6, 8) (**Figure 2**). Corticotropin-releasing hormone (CRH) is secreted by the hypothalamus in response to circadian rhythm, stress and other stimuli. CRH reaches the anterior pituitary gland *via* the portal system, where it stimulates release of adrenocorticotropin hormone (ACTH). ACTH is transported in the bloodstream and affects adrenal gland function, where it stimulates production of cortisol and its release into the circulation. Besides regulation of adrenal steroidogenesis, ACTH also acts through nonsteroidal pathways by binding and activation of melanocortin receptors to influence metabolic function and immunomodulation (16, 17). In the circulation, approximately 90% of cortisol is bound and sequestered to the corticosteroid-binding protein, and to a lesser extent (5%) albumin, with only around 5% of total cortisol existing as free ligand available for signalling (18). Given its lipophilic properties, cortisol passes relatively unimpeded through cellular membranes where it elicits signal transduction through its binding to glucocorticoid receptor isoforms. Cortisol (or other synthetic glucocorticoids) mediates negative feedback at the hypothalamus and pituitary gland to suppress further CRH/ACTH release. The homeostatic set point for this negative feedback loop is subject to modulation by circadian rhythm, severe stress and factors such as circulating inflammatory mediators.

The normal half-life of cortisol in the circulation is between 1-2 hours and is determined by several factors (19, 20). These include its direct removal from the body through terminal metabolic inactivation in the liver and renal excretion in urine (9). In this context, enzymatic clearance of cortisol in the liver involves reduction by 3α hydroxysteroid dehydrogenase and 5α reductase or 5β reductase to yield 5α-tetrahydrocortisol (5αTHF) or tetrahydrocortisol (THF) (cortisone is similarly converted to tetrahydrocortisone (THE)) (**Figure 2**). A fraction of tetrahydro-metabolites can also be further metabolized by the 20 α and 20 β reductases to α and β cortols or cortolones. Lastly, conjugation reactions of metabolites with glucuronide increase the water solubility of glucocorticoid metabolites and facilitate urinary excretion.

Further factors that influence circulating cortisol half-life include their reversable metabolism by the enzymes 11β-hydroxysteroid dehydrogenase (11β-HSD) type 1 and 2 (21) (**Figure 2**). These enzymes mediate the interconversion of active cortisol and inactive cortisone within peripheral tissues. These enzymes also allow for tissue-specific pre-receptor modulation of glucocorticoid signalling. 11β-HSD type 1 predominantly activates cortisone to cortisol in the presence of its cofactor NADPH (nicotinamide adenine dinucleotide phosphate). 11β-HSD type 1 activity is highest in metabolically active tissues including liver, fat and skeletal muscle, where it amplifies local glucocorticoid signalling (22). In contrast, 11β-HSD2 inactivates cortisol to cortisone in the presence of its cofactor NAD^+^ (nicotinamide adenine dinucleotide) and is highly expressed in the kidneys. 11β-HSD2 is considered important for preventing indiscriminate activation of the mineralocorticoid receptor (MR) by cortisol, which possesses a high affinity and agonist activity at the MR (23). At the systemic level, there is considerable continuous interconversion between cortisol and cortisone that determines a circulating equilibrium for these steroids (24, 25). Under resting conditions, a roughly 10-fold lower level of total inactive cortisone is counterbalanced by a significantly lower affinity of cortisone for circulating binding proteins, resulting in comparable levels in serum free levels as is observed with cortisol (9). Consequently, changes in 11β-HSD1 or 11β-HSD2 activity can modulate not only local tissue specific cortisol availability, but also influence systemic rates of cortisol appearance and disappearance (26–28).

## 3 Measures of systemic cortisol levels in CKD

### 3.1 Morning cortisol in blood

Early morning blood cortisol level is the most frequently reported measure of systemic glucocorticoid exposure in CKD, endeavouring to measure cortisol level at its diurnal peak. There is however considerable variability between studies in the literature (**Table 1** and **Supplementary Table S1**). No statistically significant change of morning blood cortisol levels in CKD is reported by the majority of studies published since 1995 (29–45). Still, a substantial number of studies describe significant elevation of morning blood cortisol levels with renal impairment (46–50, 52, 55), while a few isolated studies also report a significant reduction (53, 54).

**Table 1:** Morning blood cortisol levels in CKD.

Citation	Study Population	n	Finding
Studies reporting no significant difference for morning cortisol			
Asao et al. (29)	Patients with Type 2 Diabetes (eGFR >15)	77	not statistically significant (n.s.)
Karu et al. (30)	CKD stage 4	27	not statistically significant (n.s.)
Russcher et al. (31)	Haemodialysis patients & Healthy controls	18	not statistically significant (n.s.)
Raff et al. (32)	Patients on Dialysis and Healthy Controls	24	not statistically significant (n.s.)
Zoladz et al. (33)	Patients on Dialysis and Healthy Controls	43	not statistically significant (n.s.)
Deshmukh et al. (34)	Patients with CKD and Healthy Controls	11	not statistically significant (n.s.)
Oguz et al. (35)	Male Patients with CKD	60	not statistically significant (n.s.)
N’Gankam et al. (36)	Patients on Dialysis and Healthy Controls	97	not statistically significant (n.s.)
Homma et al. (37)	Patients on Dialysis, Patients with Diabetes and Healthy Controls	149	not statistically significant (n.s.)
Gunduz et al. (38)	Patients with Renal Amyloidosis and Healthy Controls	37	not statistically significant (n.s.)
Jenkins et al. (39)	Patients on Dialysis and Healthy Controls	24	not statistically significant (n.s.)
Clodi et al. (40)	Patients on Dialysis and Healthy Controls	21	not statistically significant (n.s.)
van Herle et al. (41)	Patients on Haemodialysis and Healthy Controls	11	not statistically significant (n.s.)
Morineau et al. (42)	Patients on Dialysis and Healthy Controls	60	not statistically significant (n.s.)
Letizia et al. (43)	Patients on Dialysis and Healthy Controls	34	not statistically significant (n.s.)
Vigna et al. (44)	Patients on Dialysis for >10 years & Healthy Controls	17	not statistically significant (n.s.)
Fouque et al. (45)	Patients on Dialysis and Healthy Controls	18	not statistically significant (n.s.)
Studies reporting higher morning cortisol in CKD			
Rodriguez-Gutierrez et al. (46)	Patients with CKD (stage 3 to 5D)	60	negative correlation of cortisol with eGFR (p=0.002); trend for higher cortisol with advancing CKD groups (p=0.05)
Vitolo et al. (47)	Patients with Obesity (BMI>40 & normal creatinine)	50	negative correlation of cortisol with mGFR (p=0.03)
Li et al. (48)	Patients with Hypertension (eGFR >30)	178	negative correlation of cortisol with eGFR (p=0.012); higher cortisol in low eGFR group (p=0.001)
Olsen et al. (49)	Patients with Adrenal Adenoma (eGFR >30)	164	higher cortisol with eGFR 30-60 vs eGFR >60 (p=0.02)
Afsar et al. (50)	Renal Outpatients	174	negative correlation of cortisol with creatinine clearance (p=0.015)
Armaly et al. (51)	Patients on Haemodialysis and Healthy Controls	97	higher cortisol in dialysis group (p<0.001)
Guder et al. (2006)	Patients with Heart Failure (eGFR >15)	294	positive correlation of cortisol with CKD stage (p<0.001)
Chan et al. (52)	Patients undergoing Creatinine Clearnace Testing	82	higher cortisol with CrCl <20 (p=0.002)
Studies reporting lower morning cortisol in CKD			
Arregger et al. (53)	Patients on Dialysis with Hypotension	80	lower cortisol in select subgroups of HD patients vs control group (p<0.05)
Svensson et al. (54)	Patients with Type 1 Diabetes	29	lower cortisol in reduced eGFR group (p<0.05)

eGFR, estimated glomerular filtration rate; CrCl, creatinine clearance; n.s., not statistically significant; BMI, body mass index; HD, haemodialysis.

Multivariable regression analysis to assess potential confounding influences for correlations between serum cortisol and kidney function was performed in three studies (47, 48, 55). Renal dysfunction was identified as the strongest predictor of higher serum cortisol in a cohort of patients with heart failure after adjustment for age, sex, body mass index (BMI), atrial fibrillation and diuretic use (55). Furthermore, serum cortisol was independently and negatively correlated with estimated glomerular filtration rate (eGFR) after adjustment for age, sex, antihypertensive medications, BMI, blood pressure, total cholesterol and uric acid in a cohort of patients with hypertension (48). In contrast, an independent correlation between serum cortisol and measured GFR was not apparent in the study by Vitolo et al. (47) This study however had significant methodological limitations for detecting such a relationship, as subjects with creatinine values outside the normal range were excluded from the study and the multivariable model for measured GFR included highly correlated blood urea nitrogen, renal diameter and renal plasma perfusion as independent variables besides serum cortisol.

The mixed results for early morning cortisol in CKD partly reflect limitations of this methodological approach to assess endogenous glucocorticoid physiology. The challenges relate to the volatility of morning cortisol levels and associated high measurement variability (8). Morning cortisol levels not only vary substantially from person-to-person, but also have a brief window at their peak that is technically demanding to accurately predict. Studies reporting no change in morning cortisol levels on the whole relied on smaller study cohorts (median sample size = 24, range 11 – 149), making them more susceptible to these caveats. Statistical power to detect significant changes in the context of highly variable measurements was low for many of these studies. In contrast, studies that reported significant elevations of morning cortisol with renal impairment tended to have larger study cohorts (median sample size = 130, range 50 – 294). Therefore, adequate sample size was a significant factor for the capacity of these studies to identify elevated morning cortisol in CKD.

The challenges related to determination of basal cortisol levels are made worse by lack of stringency with sample collection and laboratory assays. Even though conditions for sample collection and processing are detailed in most studies, there is no uniform procedure for all studies and hence variation in sample collection contributes to variation in results. In a minority of studies, conditions for sample collection were insufficiently stringent (33) or not reported at all (50), which severely restricts the interpretation of their results. Immunoassays were used in most studies as the laboratory technique for measuring cortisol, with only a few studies utilizing mass spectrometry-based techniques (30, 31, 36). Immunoassays have inferior specificity for cortisol and show significant inter-assay variability, meaning that this type of cortisol assay is more susceptible to interference from related metabolites and offers limited generalizability of results across studies (56).

Heterogeneity in study populations is another important reason for diverging reports on early morning cortisol in CKD. Studies describing low cortisol levels in CKD contained biases towards populations with increased risk for adrenal insufficiency. These biases included participant selection based on intra-dialytic hypotension or co-existing type 1 diabetes. Associations between high morning cortisol and low renal function were more commonly described in studies that recruited participants with specified co-existing conditions, as opposed to studies that recruited participants based on CKD alone. Co-existing conditions in these instances were severe obesity, hypertension, adrenal adenoma or heart failure (47–49, 55). Methodological differences between the two sets of studies cannot be excluded, e.g. a tendency for smaller sample sizes in studies with CKD as the principal inclusion criteria. Still, it is important to consider that morning cortisol levels are increased by renal impairment synergistically with other chronic conditions. Even at mild to moderate renal impairment, negative correlations between indices of glomerular filtration rate and morning cortisol level were evident in several cohorts with comorbidities (47–49, 55). In this context, kidney function was an independent predictor for higher morning cortisol in multivariable regression analysis in cohorts with heart failure or hypertension (48, 55). These observations are among the strongest arguments that loss of renal function is associated with higher morning cortisol. Possibly, a synergistic effect between renal impairment and other chronic health conditions with a propensity to augment cortisol makes elevations in morning cortisol more prominent.

### 3.2 Evening and diurnal cortisol in blood

**Figure 3:**
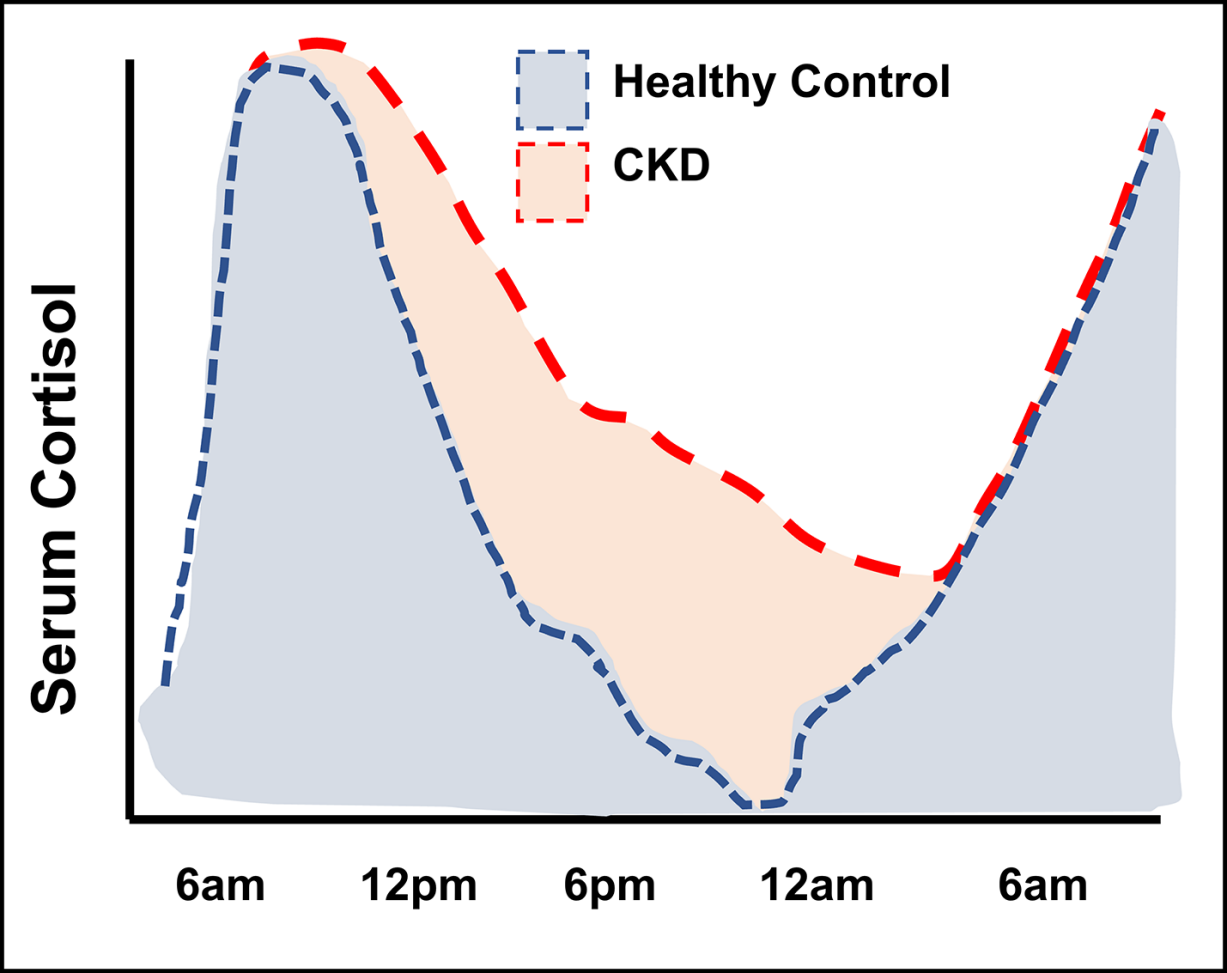
Illustration of diurnal cortisol levels in people with kidney failure compared to healthy control groups. Figure adapted from Raff et al. (32) and Deshmukh et al. (34).

Evening, or late-night cortisol measurements, taken when diurnal levels are approaching their nadir in healthy individuals, provide an additional perspective on circulating cortisol levels. It has been established that such measurements are better suited to detect elevated cortisol exposure in conditions of mild to moderate glucocorticoid excess (57). Significantly elevated levels of evening cortisol have been reported for several CKD cohorts (32, 34, 58) (**Table 2** and **Supplementary Table S2**). These studies involved patients across a range of CKD settings, including patients on renal replacement therapy. The most accurate measurements of cumulative cortisol exposure can be achieved by serial cortisol measurements over the course of the day, rather than one-off measurement in the morning or evening. Cortisol has been assessed in this way in CKD by a few small studies (31, 32, 34). The diurnal rhythm of cortisol is usually recognisable in patients with CKD, but cortisol exhibits an attenuated rate of decline throughout the day, persisting at higher levels at the nadir compared to healthy individuals. No significant association between renal function and evening cortisol levels was reported in the study by Vitolo et al. (47). However, this study excluded participants with moderate or severe renal impairment, meaning this observation is in line with other studies showing more pronounced cortisol changes from CKD stage 3 onwards (49, 59). In summary, the cumulative cortisol exposure in people with CKD appears higher over a 24-hour period, which is most apparent after the decline of the morning peak (**Figure 3**).

**Table 2:** Evening cortisol levels in CKD.

Citation	Study Population	n	Finding
Studies reporting higher evening cortisol in CKD			
Cardoso et al. (59)	Renal & Endocrine Outpatients and Healthy Controls	120	high evening cortisol in CKD 3 or 4 vs CKD 1 or control group by either diurnal measurement (p<0.05)
Raff et al. (32)	Patients on Dialysis and Healthy Controls	24	high evening cortisol in CKD 5 (HD) vs control group by either diurnal measurement (p<0.05)
Martins et al. (58)	Patients with resistant hypertension & failed dexamethasone suppression test	112	CKD more prevalent in group with 23pm salivary cortisol>3.6nM than group with cortisol<3.6nM
Deshmukh et al. (34)	Patients with CKD and Healthy Controls	11	high evening cortisol in CKD 5 vs control group (p<0.05)
Studies reporting no significant difference for evening cortisol			
Vitolo et al. (47)	Patients with Obesity (BMI>40 & normal creatinine)	50	not statistically significant (Note: proteinuria or creatinine outside normal range were exclusion criteria)
Russcher et al. (31)	Haemodialysis patients & Healthy controls	18	trend for high evening cortisol, but not statistically significant

### 3.3 Cortisol in saliva

Saliva cortisol levels are considered to represent serum free biologically active cortisol, meaning measures of salivary cortisol are less affected by variations in binding protein levels than measures of total cortisol levels in blood (56, 60). Early morning salivary cortisol in CKD has been investigated by several studies and generally no statistically significant differences were found between CKD groups and control groups (32, 42, 59) (**Supplementary Table S3**). Reduced morning salivary cortisol level was reported in one study, albeit in a cohort of dialysis patients with a selection bias for recurrent hypotension (53). In contrast, evening salivary cortisol is elevated in CKD according to two studies (**Table 2** and **Supplementary Table S2**). Cardoso et al. demonstrated higher evening salivary cortisol in patients with CKD stage 3 or 4 compared to control groups (59), while Raff et al. showed the same change for patients on haemodialysis (32). The data from salivary cortisol measurements therefore shows parallels with data from blood cortisol measurements in CKD, with higher cortisol levels being prominent in evening-time measurements.

### 3.4 Cortisol and cortisol metabolites in urine

The total amount of cortisol secreted from the adrenal glands per day can be inferred from the sum of cortisol and its metabolites excreted in urine over 24 hours. Such data is available for patients with mild to moderate CKD. Is has been shown by several studies that the total amount of glucocorticoid metabolites in 24-hour urine samples is similar to control groups (61–63) (**Table 3** and **Supplementary Table S4**). Gas chromatography-mass spectrometry was utilised in all three studies to determine cortisol metabolites in urine, representing a reliable and highly specific assay methodology. Details on urine processing, storage or addition of preservatives that may affect assay results were however not reported in the studies. Overall, the available data indicate that the total amount of cortisol secreted from the adrenal glands per day does not change in mild to moderate CKD.

**Table 3:** Cortisol and cortisol metabolite measurements in urine.

Citation	Study Population	n	Finding
Studies reporting total glucocorticoid metabolites in 24-hour urine			
McQuarrie et al. (61)	Patients with CKD or essential hypertension	100	not statistically significant
Henschkowski et al. (62)	Patients with hypertension	163	not statistically significant
Quinkler et al. (63)	Patients with CKD undergoing kidney biospy	95	not statistically significant
Studies reporting urinary free cortisol			
Chan et al. (52)	Patients undergoing creatinine clearnace testing	82	lower urinary free cortisol with CrCl <60 than CrCl >60 (p<0.001); lower urinary free cortisol with CrCl <20 than CrCl 20-60 (p<0.05)
Oguz et al. (35)	Male patients with CKD	60	lower urinary cortisol with advancing renal impairment (p=0.00001 for morning samples, p=0.074 for midnight sample)

Several putative explanations may reconcile this observation of unchanged total urinary cortisol metabolites with a pattern of increased endogenous cortisol exposure in CKD. Firstly, the control groups in all three studies examining urinary cortisol metabolites were not fully disease-free. The normal kidney function reference groups included people with treated hypertension (61, 62) or people with preserved kidney function in the context of proteinuria and/or haematuria (63). Differences in urinary cortisol metabolite excretion in CKD may be less apparent for comparisons with these specific control groups instead of control groups that are genuinely disease-free. Secondly, prolongation of cortisol half-life in circulation can account for increased endogenous exposure in CKD in the absence of increased adrenal cortisol production (see section 5). In this context, unchanged urinary cortisol metabolite amount signifies inadequate negative feedback to downregulate adrenal cortisol production and maintain healthy systemic cortisol levels. In other words, the expected compensatory finding should be reduced urinary cortisol metabolites and unchanged urine cortisol metabolite amounts represent an abnormal response. Finally, the postulated drivers for HPA axis activation are likely less prominent in early CKD, namely inflammation, acidosis and other stress factors related to chronic disease. Changes in urine cortisol metabolites may accordingly be less readily detectable in the initial phases of CKD. Unfortunately urine cortisol metabolite quantification cannot be employed to gage adrenal cortisol secretion in end-stage kidney disease given the loss of urine output in this context.

Besides excretion of cortisol metabolites, a small fraction of unchanged cortisol is also excreted in urine by the kidneys. This route of cortisol clearance has little bearing on overall cortisol clearance rates or circulating levels. Nevertheless, it is significant in clinical practise as 24-hour urinary free cortisol measurements are commonly used in the evaluation of suspected hypercortisolism. In CKD, the renal clearance of free cortisol and the absolute amount in a 24-hour urine sample are both reduced (35, 52). This means that measurement of 24-hour urine free cortisol is not a reliable test for evaluating suspected hypercortisolism in the context of CKD.

### 3.5 Cortisol protein binding

Circulating levels of free cortisol are determined by the balance between adrenal secretion, levels of corticosteroid-binding globulin (CBG) and systemic metabolism (60). Protein binding of cortisol to CBG is unchanged in CKD. CBG concentrations are comparable across CKD stages and health, cortisol binding affinity to CBG is similar in heathy and uraemic serum, and the total apparent distribution volume for cortisol is equivalent in CKD and health (19, 59, 64). This means systemic measures of cortisol can generally be compared between CKD and control cohorts without confounding by shifts in the unbound fraction of cortisol available for signal transduction.

### 3.6 Impact of dialysis on cortisol

In patients with end-stage renal failure, the life-sustaining treatment of regular dialysis can itself influence cortisol levels in addition to disease processes. Cortisol is removed from the circulation during haemodialysis (65, 66). However, this does not necessarily lead to a reduction in circulating cortisol levels. While a drop in cortisol levels from beginning to end of a haemodialysis session has been described in some studies (33, 36), other studies have reported a rise, implying increased cortisol secretory activity during dialysis (20, 43). The discrepant findings could be due to differences in patient populations or administration of dialysis. Peritoneal dialysis also removes cortisol from the circulation, but does not restore normal cortisol levels compared to healthy controls (67). Besides these acute effects, there is limited knowledge about the impact of chronic dialysis on long-term regulation of cortisol. No significant difference in morning cortisol levels were observed between patients with advanced pre-dialysis kidney failure and haemodialysis-treated patients in several studies, but group sizes were small (35, 46, 50). Further studies report no significant difference in morning cortisol levels between haemodialysis-treated and peritoneal dialysis-treated patients, but again small group sizes limit robust conclusions (40, 45). Without data on systemic cortisol levels from larger cohorts and data on diurnal cortisol levels, potential effects of long-term dialysis or dialysis modality on cortisol levels cannot be reliably evaluated.

## 4 HPA axis regulation of cortisol secretion in CKD

Circulating levels of cortisol and diurnal rhythm are determined by the finely tuned balance between adrenal secretion of cortisol under hypothalamic-pituitary control and its metabolism prior to urinary excretion. In this section we will consider research relating to the dysregulation of the HPA axis that could underpin excess cortisol exposure in CKD.

Administration of synthetic ACTH followed by measurement of serum cortisol is a method utilised to examine one aspect of HPA function and adrenal capacity to secrete cortisol. Adrenal responsiveness to ACTH is generally preserved in people with CKD, with multiple studies reporting comparable cortisol responses (35, 40, 44, 68) and one study reporting higher peak cortisol levels in renal impairment relative to control groups (46) (**Supplementary Table S5**). Close examination of findings in some of these studies may point to more sustained elevation of circulating cortisol following ACTH administration in CKD, but confirming this requires further time-course analyses (44, 46). In contrast, insufficient cortisol responses to ACTH have been reported in specific subgroups with CKD (38, 69–71). However, participant selection in these studies included biases for adrenal insufficiency (e.g. referral for adrenal insufficiency testing, history of glucocorticoid-treated glomerular disease, persistent hypotension or amyloid disease) that hamper their generalisability. Consequently, unless other risk factors for adrenal insufficiency are present, ACTH-induced cortisol release appears intact in CKD without notable differences to healthy populations.

Examinations of endogenous ACTH levels offer some further insight into HPA axis regulation at the hypothalamic-pituitary level. Levels of ACTH are variably reported as elevated or not significantly different in CKD compared to control groups. However, many of these studies rely on one-off measurements of early morning ACTH in blood. Evaluation of endogenous ACTH levels entails similar challenges as for cortisol levels, in that levels are highly dynamic over 24 hours, with many studies relying on relatively small sample sizes and significant heterogeneity in study populations. Acknowledging these limitations, comparisons of early morning ACTH level between dialysis-treated patients and control groups have found significant increases (35, 40, 41), trends for non-significant increases (39, 44) or no differences (32, 34, 43) (**Table 4** and **Supplementary Table S6**). Studies involving patients with pre-dialysis CKD tended to be larger but give a similarly mixed picture. One study involving participants from all stages of CKD reported increasing morning ACTH with loss of kidney function (35). Other studies involving participants with mild to moderate CKD and other comorbidities (severe obesity, type 2 diabetes or adrenal adenoma) identified no correlation of ACTH levels with eGFR (29, 47, 49). Diurnal rhythm of ACTH remains generally recognisable in advanced CKD, although nocturnal ACTH levels appear elevated compared to healthy volunteers according to two small studies (32, 34). Overall, a trend emerges from the reviewed literature that elevations in ACTH are more readily observed in cohorts with advanced CKD, and less evident in early stages of CKD. Consistent with this notion, metabolic acidosis, low-grade inflammation and heightened stress related to life with a chronic disease are putative drivers for increased pituitary secretion of ACTH and manifest as complications of advanced CKD (72–75). The heterogeneous distribution of inflammation and acidosis among populations with CKD may also account for observed heterogeneity in reported findings on ACTH.

**Table 4:** ACTH levels in CKD.

Citation	Study Population	n	Findings
Studies reporting higher ACTH in CKD			
Raff et al. (32)	Patients on Dialysis and Healthy Controls	24	high midnight ACTH in dialysis group (p<0.05)
Deshmukh et al. (34)	Patients with CKD and Healthy Controls	11	high evening ACTH in CKD group (p=0.04)
Oguz et al. (35)	Male Patients with CKD	60	high ACTH in CKD groups (p<0.001)
van Herle et al. (41)	Patients on Haemodialysis and Healthy Controls	11	high ACTH in dialysis group (p=0.02)
Clodi et al. (40)	Patients on Dialysis and Healthy Controls	21	high ACTH in dialysis groups (p<0.05)
Studies reporting no significant difference			
Vitolo et al. (47)	Patients with Obesity (BMI>40 & normal creatinine)	50	not statistically significant (n.s.)
Asao et al. (29)	Patients with Type 2 Diabetes (eGFR >15)	77	not statistically significant (n.s.)
Olsen et al. (49)	Patients with Adrenal Adenoma (eGFR >30)	164	n.s. (Note: age mismatch between groups)
Arregger et al. (53)	Patients on Dialysis with Hypotension	80	high ACTH in groups B or D vs control group (p<0.05); low ACTH in group C vs control group (p<0.05)
Jenkins et al. (39)	Patients on Dialysis and Healthy Controls	24	trend for high ACTH in dialysis groups (p-value not reported)
Letizia et al. (43)	Patients on Dialysis and Healthy Controls	34	n.s.
Vigna et al. (44)	Patients on Dialysis for >10 years & Healthy Controls	17	n.s. with trend for high ACTH in dialysis groups (p=0.06)

The dexamethasone suppression test is classically used to examine the negative feedback mechanism in the HPA axis, meaning the capacity to shut down adrenal cortisol secretion. Cortisol levels in CKD exhibit partial resistance to suppression by dexamethasone (a potent glucocorticoid receptor agonist with minimal mineralocorticoid activity). Evidence is robust that the standard dose of 1mg Dexamethasone given orally at midnight is less effective at supressing morning cortisol in CKD (**Supplementary Table S7**). Consistent findings are reported by numerous studies (29, 41, 49, 58, 59, 76), while only one study indicated no significant difference to the healthy control group (35). The pharmacokinetics of oral Dexamethasone appear unaltered in CKD, meaning the data reflect a genuine difference in endogenous cortisol physiology rather than an artefact related to effective Dexamethasone dose (59). When higher doses or extended courses of dexamethasone are used, a similar level of cortisol suppression can be achieved in CKD as in control groups (59, 77). This observation suggests lower hypothalamic-pituitary sensitivity to negative feedback regulation, which can be overcome at higher glucocorticoid stimulation. Another potential explanation is prolonged half-life of cortisol in CKD. Persistence of higher levels of cortisol in circulation could contribute to apparent dexamethasone resistance. Measurements of ACTH levels following dexamethasone would allow evaluation of hypothalamic-pituitary responsiveness to glucocorticoid suppression directly, but none of the available reports included such data.

## 5 Half-life, clearance and 11β-HSD metabolism of cortisol in CKD

Direct evidence for prolonged half-life of circulating cortisol is available from two studies in patients with advanced CKD (Kawai et al.: t_1/2_ = 2.9h vs. 2.1h & Wallace et al.: t_1/2_ = 1.9h vs. 0.9h for CKD and control groups) (19, 20). The kidneys are important for the clearance of cortisol in two distinct ways. These are removal of cortisol metabolites in the urine following primarily hepatic metabolism and reversible inactivation of cortisol to cortisone *via* the enzyme 11beta-hydroxysteroid dehydrogenase type 2 (11β-HSD2) (**Figure 2**) (9, 78). Several studies have examined these facets of cortisol metabolism as drivers of increased cortisol half-life in CKD.

The cortisol metabolites THF, 5α-THF and THE accumulate in patients with impaired renal function. Serum levels for these metabolites increase up to nine-fold in patients with CKD compared to control groups (36). At the same time, the total amount of THF, 5α-THF and THE in 24-hour urine samples is similar in people with moderate CKD and healthy people (see section 3.4). This means that renal clearance of THF, 5α-THF and THE is reduced in CKD, as would be expected with a reduction in renal solute clearance in general. Little research has directly investigated hepatic metabolism of glucocorticoids in CKD. A study by Deck et al. provides some evidence that the accumulation of THF, 5α-THF and THE (the end products of hepatic cortisol metabolism) may attenuate hepatic inactivation and metabolic clearance of cortisol (66). Clearance of cortisol in patients with end-stage renal failure was higher during dialysis than after dialysis, but the dialytic removal of cortisol itself accounted only for 20-35% of the difference. This suggests that dialytic removal of cortisol metabolites may enhance cortisol metabolism, through decreased inhibition by the end products of metabolism. Hence, hepatic metabolism of cortisol may be less effective in CKD.

11β-HSD enzymes mediate the reversible interconversion between active cortisol and inactive cortisone (**Figure 2**) (21). 11β-HSD1 (cortisol activating) and 11β-HSD2 (cortisol inactivating) modulate tissue-specific cortisol exposure and influence circulating cortisol half-life (24, 27). Peripheral 11β-HSD metabolism of cortisol is altered in CKD, shifting the equilibrium towards cortisol activation. On a systemic level, the balance of cortisol/cortisone interconversion by the 11β-HSD enzymes can be measured as the ratio of cortisol to cortisone, or the ratio of their respective metabolites (THF & 5αTHF to THE). This cortisol/cortisone ratio is significantly elevated in serum from patients with end stage kidney failure compared to healthy controls (36, 37, 42) (**Supplementary Table S8**). Furthermore, the cortisol/cortisone ratio in urine, or the ratio of their respective metabolites, is negatively correlated with kidney function in pre-dialysis CKD (62, 63, 79, 80). These observations demonstrate a shift towards increased serum cortisol in the equilibrium of cortisol/cortisone interconversion as renal function diminishes. On a systemic level, this contributes to a prolongation of half-life of cortisol in circulation.

Reduction of 11β-HSD type 2 activity is a determining factor for the altered cortisol/cortisone balance. Studies have shown that the kidneys are the major site for 11β-HSD type 2 expression and activity. Accordingly, cortisone production is almost abolished in anephric patients (78). Furthermore, the expression of 11β-HSD2 in kidney biopsy tissue correlates negatively with kidney function (63). This provides sound evidence that renal inactivation of cortisol to cortisone by 11β-HSD2 is impaired in CKD.

The contribution of 11β-HSD type 1 to altered cortisol/cortisone balance in chronic renal impairment is less clear. No direct measurements of 11β-HSD type 1 activity or expression in relation to impaired renal function are available in humans. It is still worth considering that elevation of 11β-HSD1 activity may occur in CKD. Inflammatory cytokines like tumour necrosis factor-α (TNFα) are elevated in CKD and are known to upregulate 11β-HSD1 activity. In line with this hypothesis, the urinary (THF+5αTHF)/THE ratio – a surrogate marker of peripheral cortisol activation – is independently correlated with the systemic inflammation marker C-reactive protein (CRP) in patients with CKD (79). Additionally, putative restoration of 11β-HSD type 2 activity through renal transplantation does not normalise the plasma cortisol/cortisone ratio or urinary ratio of THF(s)/THE (81). It is therefore possible that upregulation of 11β-HSD1 activity may occur in CKD, but further proof is required to confirm this hypothesis.

None of the studies that have examined cortisol/cortisone interconversion in CKD have considered a potential role of the co-factors NAD^+^ or NADPH for shifts in 11β-HSD enzymatic activity. CKD is generally associated with elevated oxidative stress, which is itself a driver of renal fibrosis and cardiovascular morbidity (82, 83). Endogenous changes in CKD including upregulation of NADPH oxidases would be expected to reduce the cellular pool of NADPH. However, the observed changes in CKD favouring peripheral glucocorticoid activation indicate lower oxidative 11β-HSD activity (type 2 mediated conversion of cortisol to cortisone) and possibly higher reductive 11β-HSD activity (type 1 mediated conversion of cortisone to cortisol), going against the trend of the pro-oxidative milieu in CKD. Therefore, tissue- and cellular compartment-specific redox status, besides changes in 11β-HSD type 1 or 2 expression levels, may be more important for cortisol/cortisone balance in CKD than systemic oxidative stress. Further research is required to address this question in greater detail.

In summary, the range of changes in cortisol metabolism and renal clearance in CKD synergistically cause prolongation of half-life and reduced clearance of cortisol from the circulation. Irreversible metabolic inactivation in the liver appears impeded by the accumulation of metabolic end-products, which in turn is a consequence of reduced renal filtration of these metabolites. Additionally, reversible inactivation of cortisol by 11β-HSD type 2 in the kidney is impaired. Finally, preliminary findings propose that peripheral activation of cortisol by 11β-HSD type 1 could be upregulated in CKD, although further validation is necessary. The discussed shifts in 11β-HSD enzyme activities both result in longer half-life of cortisol in circulation. Therefore, multiple mechanisms together account for the prolonged half-life of cortisol in CKD.

## 6 Summary of changes contributing to cortisol excess in CKD

Review of the literature reveals a pattern of subtle cortisol excess in CKD. This is not readily apparent from the body of evidence on morning cortisol measurements alone. Morning cortisol levels are variably reported as reduced, normal or elevated in CKD. Certain studies with larger study cohorts and arguably more robust methodology have reported negative correlations of morning cortisol with eGFR, especially in populations with additional comorbidities. Nevertheless, there are considerable methodological and conceptual limitations with morning cortisol as a marker of cortisol exposure. Importantly, further evidence supports the presence of cortisol excess in CKD. Differences to normal physiology are much more apparent in settings where cortisol is expected to be suppressed. Measurements of cortisol levels at the evening nadir or serially over 24 hours reliably demonstrate reduced rates of cortisol decline, resulting in higher evening-time cortisol levels. Furthermore, studies consistently show impaired suppression of cortisol following dexamethasone. These are hallmarks of elevated cortisol exposure in CKD.

**Figure 4:**
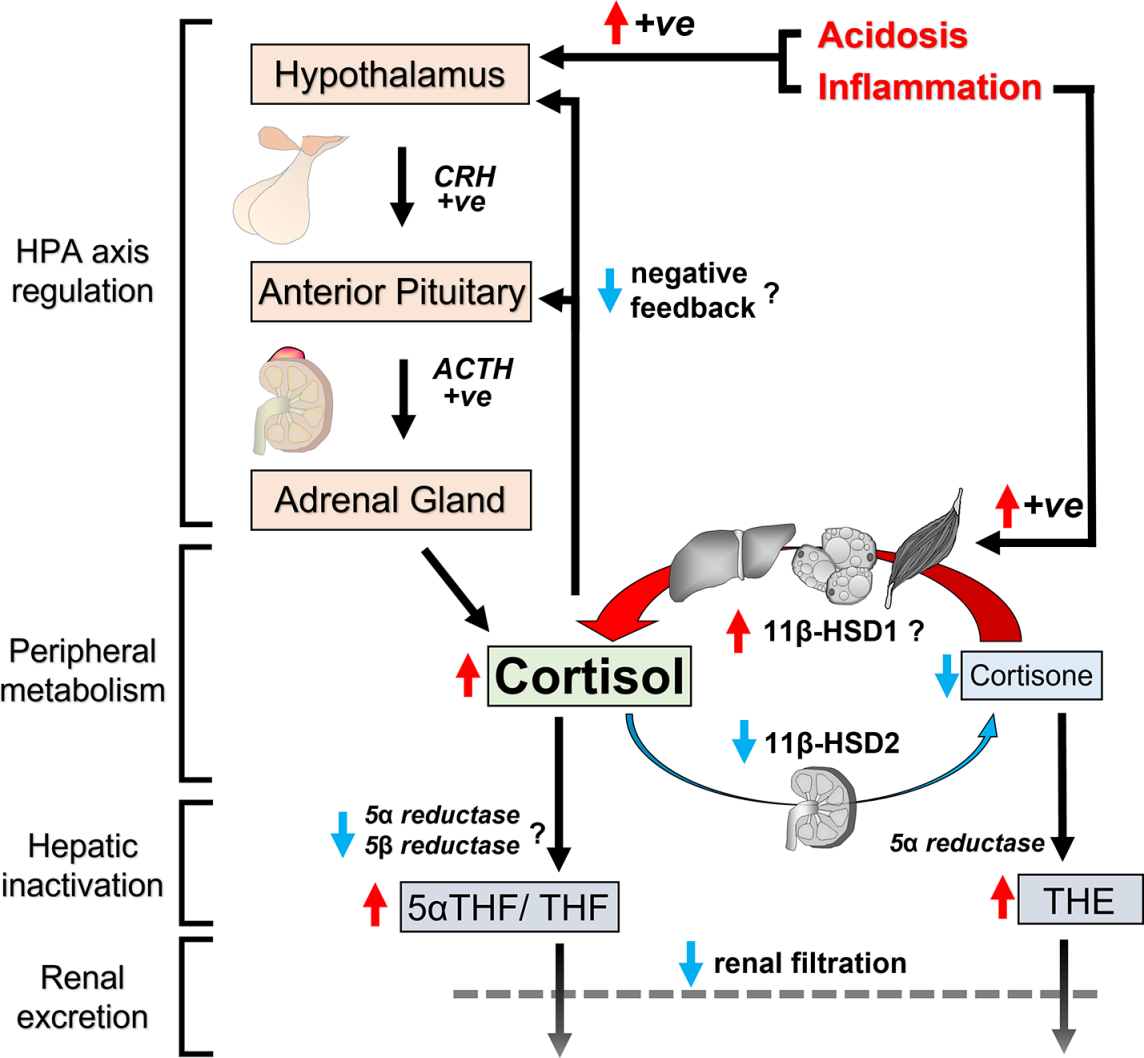
Schematic representation of changes in cortisol regulation in CKD. Cumulative exposure to circulating cortisol increases in CKD for several reasons. Clearance pathways for cortisol are impaired in CKD because reduced renal filtration causes accumulation of cortisol metabolites in circulation with postulated end-product inhibition of hepatic cortisol metabolism. Furthermore, loss of 11β-HSD2 in the kidney reduces cortisol inactivation to cortisone, contributing to prolonged half-life of cortisol in circulation. Peripheral cortisol activation by 11β-HSD1 is possibly upregulated at the same time due to increased levels of inflammatory cytokines. Inflammation and acidosis also have the capacity to induce HPA axis activation. Finally, negative feedback mechanisms in the HPA axis to suppress adrenal cortisol secretion appear to be less effective in CKD. Red arrows indicate upward changes, blue arrows indicate downward changes and question marks indicate postulated changes based on indirect evidence.

The underlying causes for cortisol excess in CKD are multifactorial, and their relative contribution is likely to vary depending on severity of CKD and co-existing morbidities (**Figure 4**). One important explanation for the observed changes is reduced clearance of cortisol. Cortisol half-life is prolonged as a result of synergistic changes directly related to renal impairment. Reduced renal filtration of cortisol metabolites and their systemic accumulation appears to impede enzymatic clearance of cortisol in the liver. Additionally, loss of 11β-HSD type 2 expression in the kidney reduces the reversible inactivation of cortisol. Dialysis treatments are not capable of fully reversing these changes.

Alongside the changes in cortisol clearance, there are probably co-existing changes in HPA axis regulation. At first glance, cumulative adrenal cortisol secretion over 24 hours appears normal in mild to moderate CKD, based on cortisol metabolite content in 24-hour urine samples. However in the context of prolonged half-life for cortisol, this observation signifies inadequate negative feedback to downregulate adrenal cortisol output and maintain healthy systemic cortisol levels. Deficiencies in negative feedback mechanisms in CKD are also supported by data from dexamethasone suppression tests. Finally, there are reports that baseline pituitary ACTH secretion is elevated in some CKD cohorts, albeit not all. Inflammation and metabolic acidosis upregulate the HPA axis and could account for variable HPA axis activation among people with advanced CKD.

All these subtle changes in endogenous cortisol regulation promote chronic cortisol excess in CKD. The pattern and chronicity of cortisol dysregulation are reminiscent of other conditions of mild cortisol excess. Even though the relative elevation of cortisol may appear modest, sustained mild cortisol excess is increasingly recognised as a risk factor for morbidity and mortality. The next section will consider evidence that cortisol dysregulation in CKD contributes to adverse outcomes.

## 7 Disturbances in endogenous cortisol regulation and their relevance for adverse outcomes in CKD

### 7.1 Survival

Glucocorticoid excess is a known risk factor for premature death (11, 13–15). Mortality in relation to cortisol levels has been investigated specifically in adults with CKD in several studies (**Table 5**). Morning cortisol was associated with adjusted all-cause mortality in two cohort studies of patients on haemodialysis (85, 87). The largest cohort study to date, involving 1255 patients with diabetes on haemodialysis, reported increased crude hazard ratios for all-cause and cardiovascular mortality with higher morning cortisol. However, these associations were not statistically significant in multivariable analysis (86). Finally, a *post-hoc* survival analysis is described by Hou et al. for a music therapy intervention in haemodialysis patients. Interestingly, there was a trend for reduced all-cause mortality and significantly lower cardiovascular mortality in the subgroup of patients with greater reduction in salivary cortisol following 1 week of music therapy compared to patients with little or no reduction in salivary cortisol (84). Taken together, the available observational evidence is generally supportive of an association between increased cortisol levels and increased mortality among patients on haemodialysis.

**Table 5:** Associations between cortisol and survival in CKD.

Citation	Study Type	Study Population	n total	Age	Male	Follow-up	At risk group	Control group	All cause mortality	Cardiovascular mortality
Kim et al. (2022)	observational - cohort study	Haemodialysis patients	133	62.9 ± 10.5 (mean, s.d.)	49%	3.3 ± 2.0 years (mean, s.d.)	above median for pre-dialysis serum cortisol (>10µg/dL)	below median pre-dialysis serum cortisol (<10µg/dL)	adjusted hazard ratio (adj. HR) 1.234 (95% C.I. 1.022-1.49, p=0.029)	not reported
Hou et al. (84)	interventional - *post-hoc* analysis in randomised controlled trial	Haemodialysis patients	49	70.5 ± 8.0 (mean, s.d.)	49%	5 years	salivary cortisol reduction <0.6pg/ml with music therapy for 1 week	salivary cortisol reduction >0.6pg/ml with music therapy for 1 week	no significant difference (p=0.051)	lower survival (63.6% vs 81.6%, p=0.028)
Gracia-Iguacel et al. (85)	observational - cohort study	Haemodialysis patients	75	64 ± 13 (mean, s.d.)	45%	1.7 years [0.7-2.6] (median, IQR)	high tertile of morning serum cortisol (≥18µg/dL)	low and middle tertile of morning serum cortisol (<18µg/dL)	adj. HR 1.16 (95% C.I. 1.027-1.309, p=0.017)	not reported
Drechsler et al. (86)	observational - cohort study	Haemodialysis patients with diabetes	1255	66 ± 8 (mean, s.d.)	54%	4 years (median)	high quartile of serum cortisol (>21.1µg/dL)	low quartile of serum cortisol (<13.2µg/dL)	adj. HR 1.10 (95% CI 0.86-1.40)	adj. HR for cardiovascular mortality 1.31 (95% CI 0.99-1.73)

Certain limitations need to be considered regarding the available literature on links between cortisol and mortality. Firstly, available reports are restricted to cohorts with end-stage renal failure. No data for patients with pre-dialysis CKD were available. Secondly, the available data is based on studies with observational design. Even though most studies conducted multivariable analyses to control for possible co-variables, confounding remains a risk in these studies. Thirdly, most studies relied on measurements of morning serum cortisol for investigating associations with mortality. It is clear from our earlier discussion that one-off morning cortisol measurements are not the most sensitive marker of endogenous cortisol changes in CKD. Furthermore, epidemiological studies have demonstrated that salivary cortisol measures or measures of diurnal variability in cortisol exhibit stronger correlations with all-cause mortality in the general population than morning blood cortisol levels (88–93). Future research should consider using these alternative markers of cortisol instead.

### 7.2 CKD progression

Glucocorticoid signalling is known to influence renal function and physiology. Short-term administration of ACTH or glucocorticoids increases glomerular filtration rate in humans, whereas patients with hypoadrenocorticism exhibit reduced glomerular filtration rate and renal blood flow (94). In specific renal diseases with underlying immune-mediated aetiology, limited courses of exogenous glucocorticoids are used as treatments to suppress inflammation and prevent injury to renal tissue (95). Exogenous ACTH has also been investigated as a therapeutic option for proteinuric glomerular diseases, with an ongoing debate about the relative significance of steroidal pathway and nonsteroidal (melanocortin) pathway signalling for mediating renal effects (16, 17, 96). Despite these insights, the consequences of altered endogenous glucocorticoid physiology and prolonged elevated glucocorticoid exposure to the kidneys are not clearly established.

Evidence in the literature that sustained hypercortisolism may adversely affect renal function is sparse and has considerable limitations. Patients with active or cured Cushing’s disease had significantly lower kidney function (measured as 24-hour creatinine clearance or MDRD-eGFR) than the matched healthy control group in a case-control study (97). Duration of Cushing’s Disease was the strongest predictor for worse GFR in multiple regression analysis. There was no evidence of increased proteinuria in the Cushing’s Disease group. In a retrospective study on patients with adrenal insufficiency, the group receiving steroid replacement therapy had a higher adjusted risk for the composite renal outcome of 40% decline in eGFR or end-stage kidney disease than the group not receiving steroid replacement therapy (98). This could suggest that prolonged glucocorticoid exposure may impair renal function, although bias between the two study groups is a significant concern. Finally, high serum cortisol after 1mg Dexamethasone suppression test was an independent risk factor for CKD in a cross-sectional study among patients with type 2 diabetes (29). No studies were available that examined links between endogenous ACTH levels and renal outcomes in CKD. In summary, the literature on any potential role of sustained hypercortisolism for progression of CKD is sparse. Information is based predominantly on study populations with primary endocrine rather than renal pathology and on study designs with high risk for bias or confounding.

Genetic association studies have reported links between genes involved in glucocorticoid homeostasis and kidney function. In a genetic association study involving a rural Chinese population, carriers of a minor allele for one of three single nucleotide polymorphisms in the 11β-HSD1 gene had lower eGFR in multivariable analysis (99). However, this association has not been confirmed in other genome-wide association studies for glomerular filtration rate (100), and no renal phenotype has been reported for the small number of adults with genetic 11β-HSD1 deficiency (cortisone reductase deficiency) described in the literature (28, 101).

Genetic 11β-HSD2 deficiency (Apparent Mineralocorticoid Excess) is well recognised to affect kidney tissue and function. Patients with homozygous lack of function mutations in 11β-HSD2 are prone to develop nephrocalcinosis and experience significant hypertension from an early age, which may lead to end-stage renal failure if untreated (26, 102). Interestingly, heterozygous carriers of a missense or frameshift mutation in the 11β-HSD2 gene also had a significantly increased risk for renal impairment compared to people with a homozygous wild-type genotype among Japanese patients with hypertension (103). The significance of non-coding or synonymous polymorphisms in the 11β-HSD2 gene for renal outcomes has been examined in three studies by two research groups. Research led by Ferrari and colleagues identified a higher prevalence of synonymous minor alleles in patients with rapid progression to end-stage renal disease or end-stage renal disease at a young age compared to other patients with end-stage renal disease (104, 105). There was however no difference in the frequency of 11β-HSD2 polymorphisms between the end-stage renal disease group as a whole compared to the healthy control group. In another study by Watson et al., a polymorphism in the 11β-HSD2 flanking micro-satellite region was more prevalent in Black patients with hypertensive end-stage renal failure than in a normotensive healthy control group (106). APOL1 genotype as a potential confounder was not reported in this study.

There is biological plausibility for sustained low 11β-HSD2 activity leading to a reduction in renal function. Low 11β-HSD2 activity in the kidney would permit indiscriminate activation of mineralocorticoid signalling by cortisol (23). Mineralocorticoid signalling in turn promotes hypertension and contributes to renal fibrosis (107, 108). However, it is more difficult to explain how synonymous or non-coding polymorphisms in the 11β-HSD2 would directly affect renal function, unless they affected 11β-HSD2 expression. Further research is needed to provide direct evidence of renal 11β-HSD2 activity as a risk factor for renal function loss in CKD outside congenital 11β-HSD2 deficiency.

### 7.3 Cardiovascular disease

Cardiovascular disease accounts for the largest share of excess mortality both in CKD and in Cushing’s syndrome. This makes it important to consider whether cortisol dysregulation in CKD contributes to cardiovascular disease burden. The circulatory and metabolic consequences of frank glucocorticoid excess that cause cardiovascular disease are well established (109). Increasingly, it is also recognised that subclinical elevations in glucocorticoid exposure promote incidence of cardiovascular disease. Such evidence comes from populations with adrenal adenoma and mild autonomous cortisol secretion, epidemiological studies in the general population and mendelian randomisation studies (15, 93, 110, 111). Of note, incomplete suppression of cortisol with 1mg Dexamethasone suppression test or blunted diurnal decline of cortisol, akin to changes seen in CKD, have been associated with cardiovascular morbidity (111–113).

Whether cortisol exposure contributes to cardiovascular morbidity specifically in CKD has not been clearly established in the literature. Diverging results are reported by two cross-sectional studies in patients on haemodialysis. Cardiovascular disease prevalence was associated with morning serum cortisol levels in the report by Kim et al. (87), but not in the report by Gracia-Iguacel et al. (85). Characteristics of the two patient groups were broadly comparable, except for higher diabetes prevalence in the study by Kim et al. Assuming the divergent findings are not by chance, it poses the question whether diabetes modifies the link between cortisol levels and cardiovascular disease prevalence or is a potential confounding factor. The only prospective study on cortisol and incidence of cardiovascular events in CKD was carried out by Drechsler et al. (86). Serum cortisol among dialysis patients with type 2 diabetes was associated with increased crude hazard ratios for myocardial infarction and composite cardiovascular events. However, these associations were attenuated in multivariable regression analysis, calling into question the significance of cortisol as a driving factor. We found no information on cortisol as a cardiovascular risk indicator for pre-dialysis CKD. Unfortunately, no data is available for cortisol markers other than morning serum levels, which would arguably be better suited to investigate correlations with cardiovascular risk in CKD.

Cumulative systemic cortisol exposure may contribute to hypertension in CKD according to two studies. The sum of 24-hour urinary cortisol metabolites correlated with ambulatory systolic blood pressure among patients with CKD stage 2-3 (114). This finding remained statistically significant after adjustment for age, BMI and eGFR. Furthermore, change in salivary cortisol levels with a music therapy intervention in haemodialysis patients correlated with changes in systolic blood pressure in a small trial by Hou et al. (84). One-off serum cortisol measurements were not correlated with hypertension in two cohorts of haemodialysis patients (85, 87), but it is important to bear in mind the inherent limitations with this marker of cortisol exposure.

Early-onset hypertension is a prominent feature in apparent mineralocorticoid excess, the congenital deficiency of 11β-HSD2 function. Despite evidence for reduction of 11β-HSD2 function in CKD, investigations on altered 11β-HSD2 activity as a risk factor for hypertension have given negative results. The ratio of urinary cortisol/cortisone or their respective metabolites was not correlated with blood pressure in two studies in pre-dialysis CKD (63, 114). Furthermore, there was no correlation between 11β-HSD2 expression in kidney biopsies and blood pressure (63). Finally, two small, blinded placebo-controlled trials of glycyrrhetinic acid, a non-specific 11β-HSD type 1 and 2 inhibitor, have been done in haemodialysis patients and found no difference in 24-hour ambulatory blood pressure (115, 116). This was despite a significant rise in plasma cortisol/cortisone ratio with the intervention.

### 7.4 Metabolic dysregulation

Insulin resistance and dyslipidaemia are evident from early stages of CKD and contribute to adverse outcomes (117, 118). Glucocorticoids act on metabolic tissues like liver, skeletal muscle and fat to promote insulin resistance and dyslipidaemia (11). Even subtle cortisol dysregulation such as elevated nocturnal levels or incomplete suppression by 1mg Dexamethasone are associated with impaired glycaemic control in the general population and in patients with adrenal adenomas (119, 120). Furthermore, several studies have implicated elevated peripheral glucocorticoid activation by 11β-HSD1 as a risk factor for type 2 diabetes (10, 121).

Current evidence on links between cortisol and impaired glucose control specifically in CKD remains inconclusive. Reduction in salivary cortisol following a music therapy intervention for patients on haemodialysis associated with a reduction in pre-dialysis glucose levels in one small trial (84). This would argue in favour of cortisol exposure influencing glycaemic control in CKD. However, further supporting evidence is limited and relies on cross-sectional studies with morning serum cortisol measurements. While a positive correlation with fasting glucose is described for a pre-dialysis CKD cohort by Afsar et al. (50), no significant association with fasting glucose or diabetes prevalence was found in a dialysis cohort by Kim et al. (87). More research is needed to examine potential links between glycaemic control and more sensitive markers of systemic cortisol exposure in CKD.

Peripheral glucocorticoid metabolism by 11β-HSD enzymes was correlated with glycaemic control in a cross-sectional analysis among patients with non-dialysis CKD stages 1-5 (79). The ratio of cortisol & cortisone metabolites in urine, a surrogate marker of systemic 11β-HSD1 activity, was associated with prevalent type 2 diabetes, and with HbA1c in the subgroup of patients with diabetes. Both associations remained statistically significant after adjustment for potential confounders in multivariable regression. A small trial of the non-selective 11β-HSD inhibitor glycyrrhetinic acid in patients on haemodialysis failed to improve markers of glycaemic control (115). As a caveat in this study, glycyrrhetinic acid and placebo were administered as part of carbohydrate-rich foods, such that both arms led to a rise in HbA1c. The lack of specificity of glycyrrhetinic acid for 11β-HSD1 represents another significant limitation, as specific 11β-HSD1 inhibitors have been shown to improve glycaemic control in patients with type 2 diabetes (122, 123). Hence, further clinical trials would be needed to explore potential benefits of 11β-HSD1 inhibition in CKD.

### 7.5 Sarcopenia and nutritional status

Loss of skeletal muscle mass and function is a prevalent complication in CKD, leading to increased rates of hospitalisation, reduced quality of life and excess mortality (124). Glucocorticoids act directly on skeletal muscle to antagonise anabolic signalling (e.g. IGF1/insulin-Akt-mTOR pathway) and activate catabolic processes (e.g. ubiquitin proteosome pathway) (125). Accordingly, measures of glucocorticoid activity predict muscle atrophy in populations with hypercortisolism (126, 127) and even in the general population (128–130). A direct role for glucocorticoid signalling in CKD-related muscle loss has been proposed by preclinical studies highlighting glucocorticoids as a required co-factor for muscle atrophy in catabolic conditions of acidosis and insulin resistance (131, 132).

The role of endogenous glucocorticoids for skeletal muscle atrophy in adults with CKD has only been investigated by a few relatively small studies. A correlation between muscle net proteolysis and plasma cortisol was reported in a study with 9 pre-dialysis CKD patients by Garibotto et al. (133). Metabolic acidosis, considered an important driver of muscle loss in CKD, was correlated with both plasma cortisol and muscle net proteolysis in univariable analysis. Interestingly, only plasma cortisol and not acidosis remained significantly associated with proteolysis in multivariable regression. This may suggest that metabolic acidosis mediates changes in skeletal muscle metabolism *via* the glucocorticoid pathway. In support of this hypothesis, observations from human experimental studies show that endogenous cortisol production is upregulated in response to acute metabolic acidosis (72, 74). However arguing against this hypothesis, muscle net proteolysis was reduced following correction of acidosis without changes in circulating cortisol in a trial with 16 patients on haemodialysis (134). Furthermore, no significant differences in lean tissue mass were found between patients with high and low morning serum cortisol levels among haemodialysis patients in a cross-sectional study (85). Given the limited available data, it therefore remains unclear to what extent endogenous cortisol changes are responsible for changes in muscle metabolism in CKD.

Information on cortisol measures in relation to nutritional status in patients with CKD is provided by a small number of studies. Patients with CKD usually experience reduced appetite and face increased risk of malnutrition, while glucocorticoids are classically considered appetite stimulants. Two cross-sectional studies in patients on haemodialysis found no significant differences in body mass index based on serum cortisol level, although there was a trend for higher adipose tissue mass in one of these studies (85, 87). Looking at 24-hour urinary glucocorticoid excretion as a marker of cumulative cortisol exposure, correlations with both body weight and body mass index were described in patients with CKD stage 2-3 by Hammer et al. (114). Deshmukh at el. conducted a small study in chronic renal failure patients to explore hormonal responses to fasting and refeeding (34). Despite elevated evening cortisol levels, patients with CKD rated their hunger sensation no higher than healthy participants during the fasting period. Also reported in the same study, cortisol or ACTH were not influenced by short-term fasting in the patient group. Lastly, no differences in cortisol or ACTH levels were found in a comparison of malnourished and well-nourished patients on haemodialysis by Jenkins et al. (39).

### 7.6 Immune dysfunction

Changes in immune function accompany CKD, characterised by inflammatory activation as well as susceptibility to infections. Systemic levels of pro-inflammatory cytokine rise with declining eGFR (75). At the same time, patients with CKD suffer higher rates and worse outcomes of common acute infectious diseases (135, 136). Inflammatory stimuli potently augment endogenous glucocorticoid signalling, both centrally through hypothalamic-pituitary upregulation of adrenal cortisol secretion and peripherally through increased glucocorticoid activation by 11β-HSD1 (73, 137). In turn, glucocorticoids exert potent immunosuppressive actions (7). This increases the risk for infection-related complications, which is a notorious problem with therapeutic glucocorticoid use (138). These interactions warrant examination whether cortisol changes in CKD are associated with immune dysfunction.

Most available studies have focussed on associations between C-reactive protein (CRP) as a marker of systemic inflammation and shifts in cortisol homeostasis. An independent association between CRP and serum cortisol was demonstrated in a cross-sectional study among 75 patients on haemodialysis, after adjustment for age, sex, previous transplant, prevalent cardiovascular disease and prevalent diabetes as potential confounders (85). A positive correlation between CRP and serum cortisol has also been reported in people with CKD stage 1-4 (50). Raff et al. investigated CRP in relation to diurnal cortisol rhythm in a small group of dialysis-dependent patients (32). Patients with disturbances in diurnal cortisol rhythm had higher CRP levels than those with normal diurnal rhythm. Links between inflammation and 11β-HSD-mediated cortisol metabolism have been examined in one cross-sectional study among patients with pre-dialysis CKD. CRP was the strongest predictor of increased peripheral glucocorticoid activation by 11β-HSD1 after adjustment for age, sex, ethnicity, kidney function, proteinuria and HbA1c (79). While an association between CRP and cortisol in CKD is described in most studies, one study among haemodialysis patients found no statistically significant difference in CRP between patients with high versus low cortisol (87).

Taken together, the available evidence suggests systemic inflammation as measured by CRP correlates positively with circulating cortisol levels and with peripheral glucocorticoid activation in CKD. Limitations in the available studies include their observational design and susceptibility to confounding. Still, there is biological plausibility for the observed correlations, given existing knowledge of HPA axis activation and 11β-HSD1 upregulation by inflammatory cytokines (73, 137). Elevated inflammatory cytokines therefore may contribute to augmented glucocorticoid exposure in CKD. The extent to which inflammation determines cortisol exposure in CKD likely varies based on level of renal impairment, co-morbidities and other factors. Otherwise, the literature contains very little information on risks relating to infection and endogenous glucocorticoid signalling in CKD.

### 7.7 Neuropsychiatric morbidity

The neuropsychiatric burden associated with CKD is increasingly recognised. The prevalence of cognitive impairment increases roughly by 12% for each 10ml/min/1.73m^2^ decrease in eGFR, representing a greater incremental risk than hypertension or hyperglycaemia (139). Additionally, the prevalence of depression is very high among patients with CKD, exceeding even prevalence rates of depression among populations with diabetes or chronic obstructive pulmonary disease (140). Glucocorticoid receptors are widely expressed in the brain, including the hippocampus, amygdala and brain cortex, structures important for cognitive and emotional function (11). Elevated cortisol stimulation causes profound and lasting disorders in affective and cognitive health (11, 141). It is therefore relevant to consider whether changes in cortisol in CKD contribute to neuropsychiatric morbidity.

Associations between cortisol levels and mood disorders in CKD have been investigated by a range of studies. An independent correlation between serum cortisol levels and Beck’s Depression Inventory score was identified in both dialysis and non-dialysis CKD patients after adjustment for multiple potential confounders by Afsar et al. (50). Conversely, two other studies did not find a statistically significant association between cortisol and depression risk in cohorts with CKD stage 4 or end-stage renal failure, but both studies were substantially smaller (30, 51). Improvements in low mood and psychological stress for dialysis-dependent patients were reported in several trials of non-pharmaceutical interventions including music therapy, bright light therapy and comedy movie watching (84, 142, 143). Interestingly, these interventions were commonly (84, 143), but not always (142), accompanied by reductions in salivary cortisol. This would be consistent with a role of cortisol for affective mood disorders in CKD, but falls short of confirming a causal link. Intriguingly, these observations also propose that non-pharmaceutical interventions have the potential to reduce cortisol exposure in patients with CKD.

The literature contains little information on cortisol and cognitive function in adults with CKD. Blood cortisol was analysed in relation to Mini-Mental State Examination scores or the cognitive function subscale of the Kidney Disease Quality of Life Short From questionnaire in two observational studies involving haemodialysis and non-haemodialysis patients (30, 50). Both studies found no significant associations.

The relationship between cortisol and sleep quality in CKD has been considered by a few studies. Morning serum cortisol levels were not correlated with Pittsburgh Sleep Quality Index (PSQI) in dialysis or non-dialysis patients (50). However, diurnal rhythm and night-time cortisol level are arguably more relevant in this context. A relatively small study by Russcher et al. reported that patients on haemodialysis frequently had abnormal diurnal cortisol rhythm and experienced worse quality of sleep compared to healthy individuals (31). Further data on the interaction between evening cortisol and sleep quality is provided by a short placebo-controlled trial of evening melatonin administration in patients on haemodialysis (144). The results show a significant improvement in PSQI, along with a reduction in salivary cortisol that is proportionally greater at night-time than in the morning. Hence, limited evidence suggests an association between evening cortisol levels and sleep quality in patients on haemodialysis, but further research is needed to confirm a causal link.

In summary, literature on the relationship between cortisol and neuropsychiatric burden in CKD contains some evidence for correlations between cortisol levels and risk of depression, but not for cognitive dysfunction. Night-time cortisol level may be associated with sleep quality in patients on haemodialysis. The evidence is largely restricted to observational data and one-off measurements of blood or salivary cortisol, representing significant limitations and precluding robust conclusions. Of note, pharmaceutical and non-pharmaceutical interventions targeted at improving mental health and sleep may have the potential to reduce cortisol levels in haemodialysis patients.

## 8 Discussion and future perspectives

The pattern of elevated cortisol signalling in CKD is characterised by blunted diurnal decline, less effective negative feedback regulation and shifts in peripheral metabolism that favour glucocorticoid activation. These features resemble other conditions with subclinical hypercortisolism, e.g. adrenal adenoma with mild autonomous cortisol secretion (15, 93, 119, 145). Studies in populations with mild autonomous cortisol secretion, as well as studies in populations with adrenal insufficiency receiving cortisol replacement therapy, epidemiologic studies in the general population and Mendelian Randomisation studies all convincingly highlight the risks associated with this subtle pattern of hypercortisolism (14, 15, 93, 110, 119, 120). Taken together, they offer clear evidence for associations with increased cardiovascular disease burden, impaired glycaemic control, prevalence of depression and increased mortality. Yet, the literature on cortisol dysregulation and adverse outcomes specifically in CKD is less well established. Several prospective studies have identified cortisol as a risk factor for mortality in patients on haemodialysis, but this was not a universal finding. Overall, studies in CKD cohorts were sparse and gave mixed results for associations between markers of cortisol homeostasis and cardiovascular morbidity, metabolic dysfunction, body composition or neuropsychiatric disease burden.

There are several limitations with the current literature on cortisol as a risk factor for adverse outcomes in adults with CKD. Firstly, the body of evidence specifically addressing this question is limited in size. Among the available studies, a considerable proportion have relatively small sample size. There is also considerable heterogeneity in the populations studied in terms of level of renal impairment and comorbidities. These factors account to some extent for mixed findings. Secondly, the majority of studies describe observational data. This research design can identify important associations between markers of cortisol homeostasis and complications of CKD, but it precludes reliable conclusions about causal relationships. Observational studies carry higher risk for confounding and bias, which some studies have attempted to address through statistical adjustment for covariates. Thirdly, studies commonly rely on one-off measurements of cortisol. Yet, this marker is not very sensitive for the changes in endogenous glucocorticoid signalling that occur in CKD. Additionally, research in non-CKD populations clearly illustrates that morning blood cortisol levels are less strongly associated with adverse outcomes of mortality and cardiovascular risk than diurnal cortisol decline or dexamethasone resistance (15, 57, 93, 145).

Based on the current literature, there are several recommendations for future research on the role of endogenous glucocorticoid signalling in CKD. For research focussing on shifts in cortisol homeostasis as a risk factor for adverse outcomes, it is important to move beyond one-off measurements of blood cortisol levels. Instead, investigators should consider markers of cortisol diurnal rhythm, dexamethasone suppression testing and peripheral metabolism of cortisol by 11β-HSD enzymes. There is stronger evidence that these markers are deranged in CKD. There is also clear evidence that these markers associate with clinically relevant outcomes for non-CKD populations. To enhance diagnostic specificity with the standard overnight 1mg dexamethasone suppression test, concomitant measurement of serum dexamethasone levels to confirm adequate suppressive levels is recommended (146, 147). Sampling of salivary cortisol levels has also been validated in populations with impaired renal function (32). This may represent a more convenient strategy for repeat sample acquisition or out-of-office testing than blood tests. Salivary cortisol measurements also remain viable in patients with end-stage renal failure, where glucocorticoid measures in urine are not feasible. Finally, the evidence for cortisol as a risk factor in CKD will be strengthened by collecting longitudinal rather cross-sectional data.

For research aiming to evaluate the potential therapeutic benefits of manipulating endogenous glucocorticoid homeostasis in CKD, a range of strategies could be attempted. It is known that CKD entails metabolic acidosis and subclinical systemic inflammation, and that these conditions can activate the HPA axis. Hence, future trials could examine how effectively interventions to limit metabolic acidosis or subclinical systemic inflammation reduce cumulative cortisol exposure in CKD. Furthermore, the literature offers examples of non-pharmaceutical interventions that have reduced measures of cortisol in short-term trials. Larger studies with longer follow-up durations would be needed to evaluate the potential of non-pharmaceutical and life-style interventions to restore normal parameters of cortisol homeostasis in CKD. Finally, pharmaceutical inhibitors of 11β-HSD1 are available and have demonstrated a good safety profile in Phase II clinical studies (122, 123). 11β-HSD1 inhibitors have the potential to reduce the glucocorticoid actions in metabolic tissues like liver, fat and skeletal muscle. Blockade of peripheral glucocorticoid activation may also counteract the prolonged half-life of circulating cortisol in CKD. Given the documented changes in glucocorticoid homeostasis and metabolism by 11β-HSD enzymes in CKD, there may be a therapeutic niche for 11β-HSD1 inhibitors in this patient population.

To conclude, a state of subclinical hypercortisolism develops in CKD because of impaired clearance of cortisol, shifts in peripheral cortisol metabolism by 11β-HSD and postulated upregulation of the HPA axis. Characteristic features include blunted diurnal decline of cortisol levels and incomplete cortisol suppression with low dose dexamethasone. Such changes in cortisol homeostasis are associated with higher mortality, cardiovascular disease burden, impaired metabolic function and neuropsychiatric morbidity according to studies in non-CKD populations. Even though some studies report consistent associations between markers of cortisol and adverse outcomes in CKD, the evidence generally is less well established in the context of CKD specifically. Further research is needed to validate links between disturbances in endogenous glucocorticoid function in adults with CKD and adverse outcomes, and to test whether therapeutic interventions to correct or reverse these changes can lead to clinical benefit.

## Author contributions

Conceptualization: MS, LH, RH. Literature search: MS. Writing – original draft: MS. Writing – review and editing: MS, LH, RH. All authors contributed to the article and approved the submitted version.
